# Silencing of MicroRNA-21 Confers Radio-Sensitivity through Inhibition of the PI3K/AKT Pathway and Enhancing Autophagy in Malignant Glioma Cell Lines

**DOI:** 10.1371/journal.pone.0047449

**Published:** 2012-10-15

**Authors:** Ho-Shin Gwak, Tae Hoon Kim, Guk Heui Jo, Youn-Jae Kim, Hee-Jin Kwak, Jong Heon Kim, Jinlong Yin, Heon Yoo, Seung Hoon Lee, Jong Bae Park

**Affiliations:** 1 Specific Organs Cancer Branch, Research Institute and Hospital, National Cancer Center, Goyang, Korea; 2 Cancer Cell and Molecular Biology Branch, Research Institute and Hospital, National Cancer Center, Goyang, Korea; University Hospital Hamburg-Eppendorf, Germany

## Abstract

Radiation is a core part of therapy for malignant glioma and is often provided following debulking surgery. However, resistance to radiation occurs in most patients, and the underlying molecular mechanisms of radio-resistance are not fully understood. Here, we demonstrated that microRNA 21 (miR-21), a well-known onco-microRNA in malignant glioma, is one of the major players in radio-resistance. Radio-resistance in different malignant glioma cell lines measured by cytotoxic cell survival assay was closely associated with miR-21 expression level. Blocking miR-21 with anti-miR-21 resulted in radio-sensitization of U373 and U87 cells, whereas overexpression of miR-21 lead to a decrease in radio-sensitivity of LN18 and LN428 cells. Anti-miR-21 sustained γ-H2AX DNA foci formation, which is an indicator of double-strand DNA damage, up to 24 hours and suppressed phospho-Akt (ser473) expression after exposure to γ-irradiation. In a cell cycle analysis, a significant increase in the G_2_/M phase transition by anti-miR-21 was observed at 48 hours after irradiation. Interestingly, our results showed that anti-miR-21 increased factors associated with autophagosome formation and autophagy activity, which was measured by acid vesicular organelles, LC3 protein expression, and the percentage of GFP-LC3 positive cells. Furthermore, augmented autophagy by anti-miR-21 resulted in an increase in the apoptotic population after irradiation. Our results show that miR-21 is a pivotal molecule for circumventing radiation-induced cell death in malignant glioma cells through the regulation of autophagy and provide a novel phenomenon for the acquisition of radio-resistance.

## Introduction

Glioblastoma multiforme (GBM), the most common primary malignant brain tumor, has a poor prognosis. Radiation therapy is one of the standard treatment modalities for GBM, consisting of concomitant chemo-radiotherapy with temozolomide after debulking surgery [1]. Although radiation has been used in practice, it remains poorly understood how radio-resistant cancers survive after radiation injury, and developing ways to enhance or increase radio-sensitivity have been limited [2]. The difficulties identifying a radiation sensitizer or adjuvant might be attributed to the complex genetic cellular response to radiation. Previous studies have observed that the expression of various genes, which are involved in apoptosis, the cell cycle, and p53 pathways, change during the early phase following irradiation [2]–[6]. These results suggest that a given radio-sensitizer might need to simultaneously regulate multiple genes to sensitize a response to radiation.

MicroRNAs are small non-coding, endogenously encoded, single-stranded RNAs of about 22 nucleotides in length that direct the complex regulatory networks of animals and plants by targeting mRNAs for cleavage or translational repression [7], [8]. MicroRNAs are deeply involved in resistance or sensitization to anti-cancer drugs or radiation [2], [9]. Therefore, we hypothesized that onco-microRNAs could be involved in overcoming radiation-induced cell injury. miR-21 is significantly elevated in GBM and malignant glioma cell lines [10]. The effect of miR-21 is related to various cellular responses including anti-apoptotic events, tumor growth, and chemo-resistance [10]–[16]. Down-regulation of miR-21 leads to repression of the anti-apoptotic effects in glioma. Up-regulation of miR-21 is triggered in glioma cells lacking functional phosphatase and tensin homolog (PTEN), but not in those harboring wild-type PTEN, and is responsible for glioma invasion by disrupting the negative feedback circuit of Ras/MAPK signaling mediated by Spry2. Furthermore, miR-21 up-regulation is observed in most malignant glioma tissues of patients. Based on these studies, we evaluated, here, whether miR-21 is associated with the radio-resistance of glioma cells. If miR-21 contributes to radio-resistance, antisense miR-21 could lead to radio-sensitization of glioma cells.

Among the complicated molecular responses to radiation in cancer cells, activation of the RAS/PI3K/AKT pathway results in resistance to radiation therapy[17]–[19] and synthetic PI3K inhibitors radio-sensitize some cancer cells including malignant glioma[20]–[22]. Apoptosis after irradiation is typically delayed in some radio-resistant cancer cells via transition at the G_2_/M cell cycle phase[23]–[25] and autophagy is observed in radiation-damaged cells including malignant glioma cells, although whether this is protective against or catastrophic to cell death remains inconclusive [26], [27]. Thus, we examined the influence of anti-miR-21 on these radiation-induced cellular responses as possible mechanisms of the anti-miR-21 induced radio-sensitization observed in our study.

## Results

### Radio-resistance and miR-21 Expression

**Figure 1:**
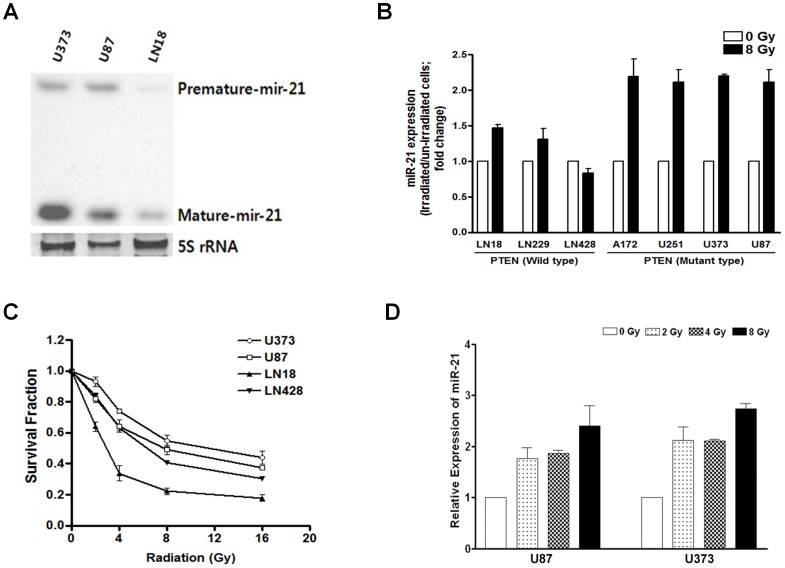
Endogenous and radiation-induced microRNA 21 (miR-21) expression. (A) Northern blotting of endogenous miR-21 in U373, U87, and LN18 glioma cell lines without irradiation. (B) After exposure to the indicated dose (8 Gy) of γ-irradiation, radiation-induced increased miR-21 expression was observed in both phosphatase and tensin homolog (PTEN) wild type and deficit glioma cell lines. Real-time PCR data is plotted as relative expression compared to the endogenous level of each control. (C) Cell survival curve after irradiation shows that the observed radiosensitivity reversely correlated with miR-21 expression measured previously. (D) The relative expression of miR-21 after γ-irradiation in U373 and U87 cells is illustrated by real-time PCR data and shows a dose-dependent increase. Each error bar indicates the standard deviation of three independent experiments.

First, we observed endogenous miR-21 expression in the various glioma cell lines, which were available at our laboratory, and increased miR-21 expression was observed by qRT-PCR in response to irradiation (2 hours after 8 Gy, Figure S1). Three cell lines that were different in both miR-21 expression levels and PTEN status (i.e., U87 and U373 of PTEN deficit, LN 18 of PTEN wild type) were chosen to confirm endogenous expression of the mature form of miR-21 using Northern blot (Figure 1A). The relative expression level of miR-21, compared to endogenous level of miR-21 in LN 18 cells as a control, was 5.5 (±1.3, standard deviation) in U87 cells and 31.3 (±7.9) in U373 cells (Figure S1). After 8 Gy irradiation, the miR-21 level increased up to 2.0 fold or more compared to the endogenous level in PTEN mutant glioma cells (2.1 fold in U87 cells, and 2.2 fold in U373 cells), while 1.4 fold or less in PTEN wild type glioma cells (1.4 fold in LN 18 cells and 0.87 fold in LN 428 cells) (Figure 1B). A cytotoxic cell survival curve was obtained after irradiation of the four cell lines to radiation doses of 2–16 Gy. The results showed that radio-resistance between the four glioma cell lines was in accordance with expression of miR-21 after irradiation (Figure 1C and Figure S1). To confirm the radiation-induced increase in miR-21 expression, we examined miR-21 expression by real-time PCR in a range of radiation doses provided by the cytotoxic cell survival assay at 2 hours after γ-irradiation. A dose-dependent elevation in miR-21 expression was confirmed by real-time PCR following γ-irradiation in U87 and U373 cells (Figure 1D).

### Suppression of Radio-resistance by Anti-miR-21

**Figure 2:**
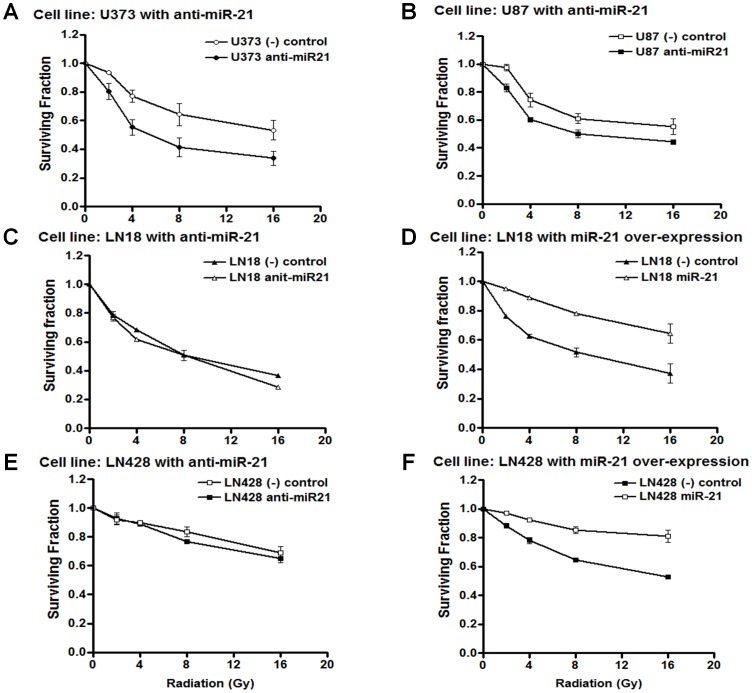
The radio-sensitivity of glioma cells according to the modulation of microRNA 21 (miR-21). Anti-miR-21 (100 nM) or negative control (100 nM) was transiently transfected into U373 and U87 cells for loss-of-function. miR-21 (1 µg) or control plasmid (1 µg) was transiently transfected into LN18 and LN 428 cells for gain-of-function. The cells were plated onto 96 well microplates and incubated for a further 48 hours prior to γ-irradiation. After exposure to the indicated level of ionizing irradiation, the surviving cell fraction was measured using a sulforhodamin-B (SRB) assay at 5 days, and the effect of miR-21 modulation is illustrated for each cell line (A–F). Each error bar indicates the standard deviation of three independent experiments.

To test whether anti-miR-21 could radio-sensitize the cells, we silenced miR-21 using anti-miR-21 and measured its influence on radio-sensitivity using the cytotoxic cell survival assay. After anti-miR-21 was introduced into U373 and U87 cells, we confirmed miR-21 down expression using real-time PCR (Figure S2). Anti-miR-21 sensitized the U373 glioma cells to radiation in proportion to the radiation dose given; 0.86 of the surviving fraction relative to the negative-control transfected at 2 Gy, 0.71 at 4 Gy, and 0.64 at 8 Gy in cytotoxic cell survival assay (Figure 2A). The surviving fraction of silenced mir-21 in U87 cells was 0.85 at 2 Gy and 0.82 at 8 Gy (Figure 2B). Furthermore, in LN 18 and LN 428 cells, which showed lower miR-21 expression level compared with PTEN mutant U87 and U373 cells, the radio-sensitization effect of anti-miR-21 was not observed (Figure 2C and E). These results showed a correlation between miR-21 expression level and the radio-sensitization effect of anti-miR-21 and suggest a dependency of radio-resistance on miR-21.

To confirm the role of miR-21 in radio-resistance, we tested the effect of miR-21 over-expression in LN18 and LN 428 cells. miR-21 over-expression was confirmed by Northern blot (Figure S2C), and the elevated miR-21 expression led to the radio-resistance of LN 18 and LN 428 cells compared to that in the negative control in proportion to the radiation dose given: 1.25 and 1.10 of the surviving fraction relative to the negative-control transfected at 2 Gy, 1.42 and 1.18 at 4 Gy, 1.51 and 1.32 at 8 Gy, and 1.73 and 1.53 at 16 Gy, respectively (Figure 2D and F). These effects of miR-21 knockdown in U373 and U87 cells, and miR-21 over-expression in LN 18 cells were again confirmed by the colony forming assay (Figure S3). Taken together, we concluded that suppressing miR-21 decreased radio-resistance of the malignant glioma cell lines tested.

### Delayed Resolution of DNA Damage Foci by Blocking miR-21

**Figure 3:**
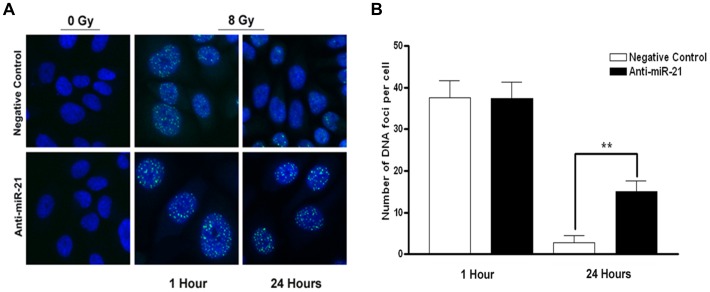
Observation of γ-H2AX in anti-microRNA 21 (miR-21)-treated U373 cells. A positive γ-H2AX signal was observed at 1 hour after γ-irradiation in both groups (A; top and bottom of middle panels), whereas its signal persisted up to 24 hours in anti-miR-21-transfected cells (A; bottom of right panel) unlike the negative control transfected cells (A; top of right panel). A quantitative comparison of DNA foci revealed that miR-21 silencing significantly inhibited the repair of DNA damage in U373 cells (p<0.001) (B). DNA foci were counted in up to 300 cells. γ-H2AX and nucleus were labeled with Alexa 488 (FITC; green) and DAPI (blue).

We further investigated whether suppressing miR-21 would influence the resolution of DNA double-strand breaks (DSBs) induced by irradiation. We observed accrual of γ-H2AX to the DNA DSB foci as an indicator of radiation-induced DNA damage. Our tested cells contained no foci in the nuclei in the absence of irradiation (Figure 3A). U373 cells transfected with anti-miR-21 were irradiated, and the number of DNA foci representing the amount of unrepaired DSBs was counted and compared to that of cells transfected with the negative control (Figure 3A and B). Radiation-induced γ-H2AX foci were readily detectable at 1 hour of irradiation in both the anti-miR-21 and negative-control treated groups, but without significant differences in the amounts (Figure 3B). Interestingly, adding anti-miR-21 led to a substantial percentage of cells retaining these foci for up to 24 hours such as 14.6% (±2.7, standard deviation) in the anti-miR-21-treated group and 2.8% (±1.7) in the negative-control treated group. The difference in the percentage of cells retaining DNA foci between the two groups was significant (*p*<0.01).

### Suppression of PI3K/Akt Signaling Pathway by Anti-miR-21

**Figure 4:**
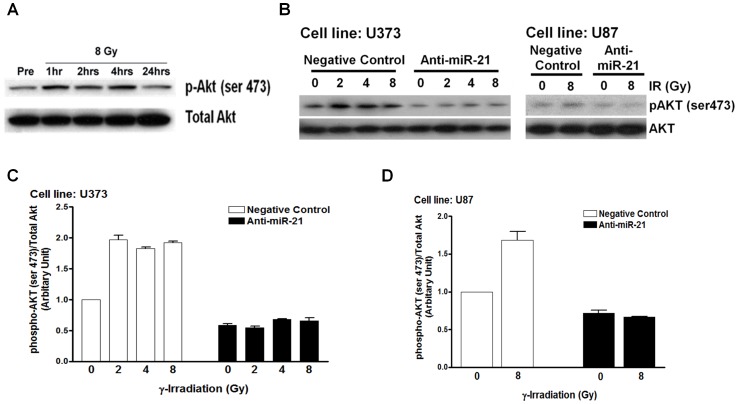
Suppression of γ-irradiation-induced phospho-Akt upregulation by antimicroRNA 21 (miR-21) in glioma cells. (A) Phospho-Akt (ser 473) expression was measured by Western blot at the indicated time intervals after γ-irradiation in U373 cells. Phospho-Akt (ser 473) expression at 4 hours after γ-irradiation showed a dose-dependent increase in the negative-control treated group and suppression in the anti-miR-21-treated U373 and U87 cells in both the Western blot (B) and the Image software analysis of their intensities (C and D, respectively). Each bar on a column represents the standard error of the mean for triplicate experiments.

Because the PI3K signaling pathway is usually activated in glioblastoma, and inactivation of this pathway impairs DNA repair following γ-irradiation [31], [32], we examined whether anti-miR-21 would affect Akt phosphorylation of PI3K downstream. The endogenous level of phospho-Akt expression (ser 473) in U373 cells was up-regulated at 1, 2, and 4 hours after γ-irradiation compared to that in the non-irradiated control and was normalized to the control level at 24 hours after γ-irradiation (Figure 4A). According to the results of Kao et al. [32], treating malignant glioma cells (U251) with LY294002, which is a PI3K inhibitor, results in impaired DNA repair, as measured by γ-H2AX staining, and we confirmed the same effect in U373 cells (Figure S4A). Therefore, we assumed that the elevated phospho-Akt expression (ser 473) at the early or mid-phase after γ-irradiation could mediate the repair of DNA damage, and that the delayed DNA damage by anti-miR-21 in our study may have resulted from inhibiting PI3K/Akt signaling. To test our assumption, we investigated whether anti-miR-21 could suppress the increase in phospho-Akt (ser 473) expression induced by γ-irradiation. Phospho-Akt (ser 473) expression measured by Western blot at 4 hours after γ-irradiation increased in a dose-dependent manner in the negative control-treated group, whereas its expression was suppressed in the anti-miR-21-treated group in both U373 and U87 cells (Figure 4B). To confirm our observation in a quantitative manner, each blot was analyzed for intensity using image analysis software (Photoshop ver. 7.0, Adobe). The phospho-Akt protein expression level, standardized to total Akt level, after irradiation was again confirmed to increase in a dose-dependent manner, whereas anti-miR-21 treatment dampened the increase (Figure 4C and D). Rad51, another DNA DSB repair protein affected by Akt activation, was also examined whether it would be influenced by anti-miR-21. Western blot showed elevated expression of Rad51 after irradiation in negative control, whereas it was totally abolished in anti-miR-21 treated in U373 cells (Figure S4 B and C).

To know if anti-miR-21 mediated inhibition of Akt phosphorylation would be vice versa with miR-21 over-expression in PTEN wild type cells, at which increased radio-resistance was observed, we observed phospho-Akt level in miR-21 over-expressed LN428 cells after irradiation with Western blot. As expected Akt phosphorylation after irradiation was minimal (<1.2 fold) in LN428 cells and mir-21 over-expression did not increased the phospho-Akt level (Figure S5A and B). To evaluate if radiosensitivity induced by anti-miR-21 or radio-resistance acquired by miR-21 over-expression were further affected by phospho-Akt inhibition with LY294002, we performed cell survival test with or without LY294002. In U373 (PTEN non-functional, miR-21 over-expressing) cells, LY294002 showed anti-apoptotic effect in the control group but revealed ‘a little effect on radiosensitivity’ in miR-21 knock down cells (Figure S6A). In LN428 (PTEN functional, miR-21 not-over-expressed) cells, LY294002 did not show any discernible difference of radiosensitivity either in the control groups or in the miR-21 over-expressed groups (Figure S6B). Taken together, these results suggest that miR-21 induce radio-resistance in both AKT-dependent and independent pathway.

### Increase in the G_2_/M Population by Anti-miR-21

**Figure 5:**
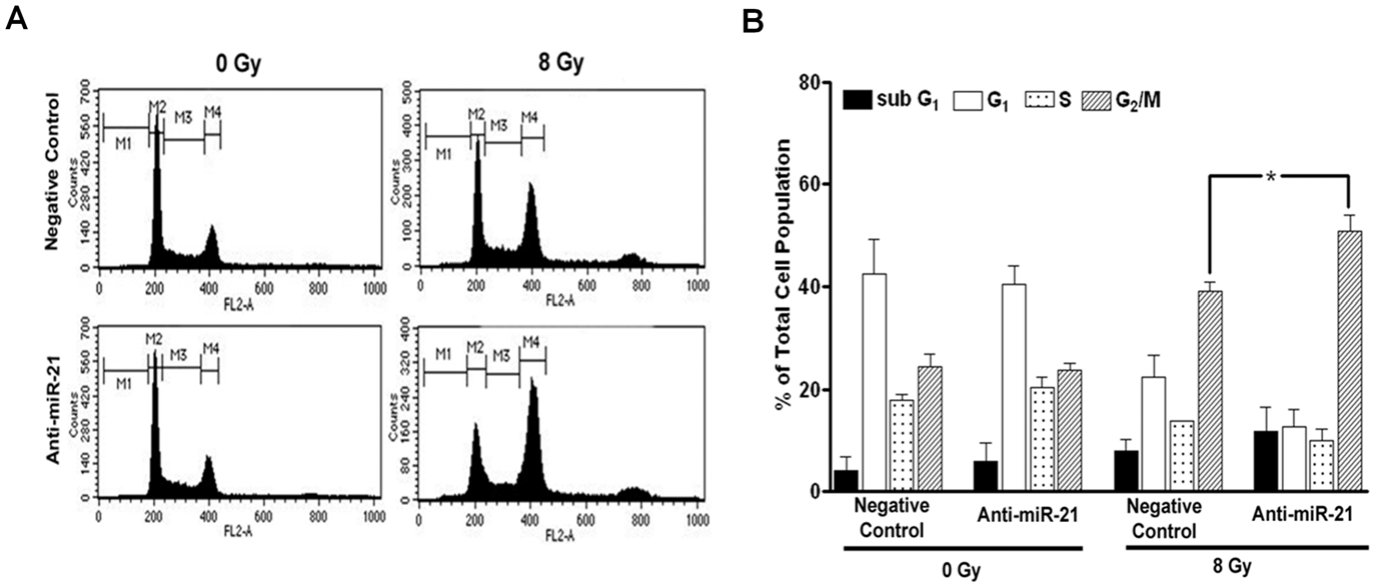
Cell cycle change after γ-irradiation in anti-microRNA 21 (miR-21)-treated U373 cells. (A) An illustrative case showing cell cycle changes 48 hours after exposure to 8 Gy radiation. (B) Statistical analysis of independent triplicate experiments showed significant differences between the negative-control and the antimiR-21 treated cells in terms of the G_2_/M population (*p<0.05), but the subG_1_ population representing apoptosis failed to reach statistical significance.

To test whether the effect of miR-21 on radio-resistance could affect cell cycle arrest after γ-irradiation, we performed a cell cycle analysis and compared the differences in the cell cycle population between the negative-control and anti-miR-21-treated U373 cells after γ-irradiation. As expected, the cell cycle analysis at 48 hours after γ-irradiation (8 Gy) revealed an increase in the G_2_/M population in both the negative-control and anti-miR-21-treated groups (Figure 5A). We analyzed the results from three independent experiments to quantify the difference in the G_2_/M population between the control and anti-miR-21-treated groups (Figure 5B). Although an increase in the G_2_/M population was common to both the negative-control and anti-miR-21-treated groups, miR-21 silencing resulted in a significantly higher G_2_/M population of 50.8% (±3.2, standard error) compared to that in the control group (39.0±2.0%) (*p = *0.04, unpaired *t*-test). Additionally, the subG_1_ population (representing apoptosis) apparently increased but was statistically insignificant in the anti-miR-21-treated group (11.8±2.7%) when compared to that in the control group (8.0±1.2%).

### Increase in Autophagy and Early Apoptosis by Anti-miR-21

**Figure 6:**
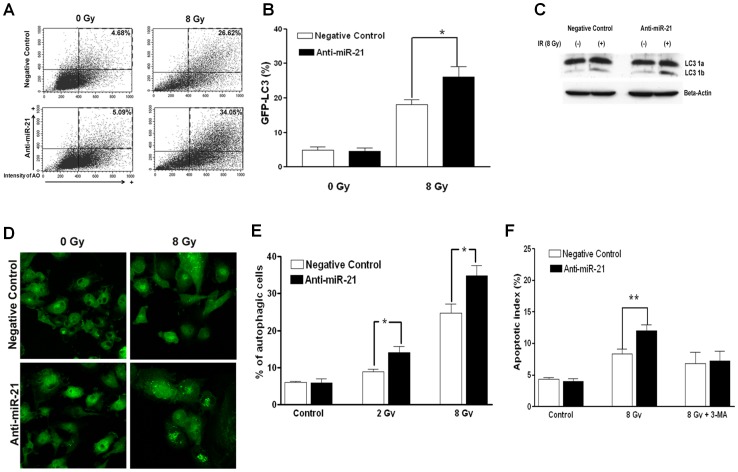
Measurement of autophagy and early apoptosis (Annexin-V) in anti-microRNA 21 (miR-21)-treated U373 cells. (A) A representative illustration showing the flow cytometry measurement of acidic vesicular organelles (AVOs) by acridine orange staining, representing autophagic activity 48 hours after exposure to 8 Gy of radiation. (B) Statistical analysis of independent triplicate values of the percentage of cells harboring AVO resulted in significant differences between the negative-control and anti-miR-21-treated cells (*P<0.05). (C) Western blot of the LC-3 protein, in which the appearance of the lower 1b band represents autophagosome-lysosome fusion. (D) Green fluorescent protein-labeled LC-3 protein (GFP-LC3) was stably over-expressed in U373 cells using a lentiviral vector, and GFP-LC3 granule-positive cells after irradiation (white arrow) were counted using a fluorescence microscope (10×20 high power fields; HPF). (E) The number of GFP-LC3 granule positive cells was counted as a percentage of total observed cells. Statistical analysis of the average percentage of ten HPFs showed significant differences (*P<0.05). (F) The flow cytometry measurements of Annexin-V staining representing apoptotic indices. The increased early apoptotic index 48 hours after 8 Gy of irradiation in anti-miR-21-treated U373 cells compared to that in negative control-treated cells (**p<0.01). Augmented apoptosis by transfection of anti-mir21 was neutralized by a 48 hour incubation with 10 mM 3-MA, an autophagy inhibitor. Each bar on a column represents the standard error of the mean for triplicate experiments.

Although it is still unclear whether radiation-induced autophagy is advantageous or disadvantageous to cell death after irradiation, autophagy can be measured by acidic vesicular organelles (AVO) after irradiation of malignant glioma cells [26], [27]. Other studies have shown a cell cycle change in the accumulated G2/M population after irradiation in solid, radio-resistant cancer cells [23]. Therefore, we postulated that the significant increase in the G2/M population in anti-miR-21-treated cells, might be related to radiation-induced autophagy. First, we measured autophagy by AVO formation, as stained by acridine orange. Flow cytometric measurements of AVO at 48 hours after irradiation (8 Gy) revealed significantly increased autophagy in anti-miR-21-treated U373 cells compared to that in negative-control treated cells (Figure 6A). An analysis of independent triplicate values of the autophagic index (the percentage of AVO-positive cells) showed an increase in autophagy after γ-irradiation, and anti-miR-21-treated cells showed significantly enhanced autophagic indices at both 2 and 8 Gy compared to those in the corresponding negative control (p<0.05, unpaired *t*-test; Figure 6B).

To confirm autophagy after irradiation under the influence of anti-miR-21, we assayed the LC-3 protein, in which the appearance of the lower 1b band represents autophagosome-lysosome fusion. An increased level of LC-3 1b protein was obvious in anti-miR-21-treated cells compared to that in the negative control (Figure 6C). Additionally, GFP-LC3 was stably expressed in U373 cells to visualize autophagy. During the autophagic process, LC3 is concentrated in autophagosome membranes, and fluorescent GFP-LC3 spots can be used to monitor autophagy [33]. The number of cells with GFP-LC3 spot fluorescence was averaged from 10 low power fields (×200), in which 24–63 GFP bearing cells were observed. The percentage of autophagic cells increased in the anti-miR-21-treated cells at 48 hours after γ-irradiation (8 Gy) compared to that in the negative-control treated cells (p<0.05, Figure 6D and E).

Although our cell cycle analysis did not show a statistically significant increase in the subG_1_ population after γ-irradiation (Figure 5B), we assumed that autophagy augmented by anti-miR-21 could contribute to early apoptosis at 48 hours after irradiation. As a result of the experiment, apoptotic indices measured by Annexin-V of early apoptosis marker, increased moderately after γ-irradiation (8 Gy) (12.0±0.9% for anti-miR-21-treated cells and 8.3±0.8% for the negative control; Figure 6F, middle column). The difference in the indices between the anti-miR-21- and the negative-control treated cells was statistically significant (*p*<0.05, unpaired *t*-test). After confirming the inhibitory effect of 3-methyl adenylate (3-MA) on radiation-induced autophagy (Figure S7A), we evaluated if 3-MA could block enhanced early apoptosis induced by anti-miR-21. The early apoptotic population after γ-irradiation in the anti-mir-21-treated cells were neutralized to the level of the negative-control treated cells by inhibiting autophagy with 2 mM 3-MA (7.3±1.6% for anti-miR-21-treated cells and 6.8±1.8% for the negative control; Figure 6F, right column).

To evaluated if anti-miR-21 mediated increased autophagy was solely dependent on inhibition of Akt phosphorylation or not, we examined the difference of autophagic index between anti-miR-21 treated cells and anti-miR-21 plus LY294002, which is known to be completely blocked Akt phosphorylation. Autophagy after irradiation was increased with LY294002 and further increased when combined with anti-miR-21 (Figure S7B). And in another attempt, we observed a change of autophagy in LN 428 cells with miR-21 over-expression, at which the phosphorylation of Akt was not incerased. However, miR-21 over-expression showed significant increase of autophagy after irradiation compared to negative-control treated (Figure S7C). These results suggested that anti-miR-21 can affect autophagy and partly cell death after irradiation other than inhibition of Akt phosphorylation.

## Discussion

Our results demonstrated that miR-21, which plays an anti-apoptotic role in cancers, could be a target molecule for radio-sensitization of glioma cells. We observed radiation-induced expression of the mature form of miR-21, and the level of expression correlated with radio-resistance measured by a cytotoxic cell survival assay of the tested cell lines. Furthermore, anti-miR-21 showed radio-sensitization in PTEN-deficit cell lines (U87 and U373 cells) and miR-21 overexpression lead to radio-resistance in the PTEN functional cell line (LN 18 and LN 428 cells). Anti-miR-21 suppressed radiation-induced phospho-Akt expression and enhanced autophagy in irradiated cells. These results suggest that miR-21 might regulate radio-resistance by modulating autophagy.

### miR-21 Affects Resistance to Radiation in Malignant Glioma Cells

We recently demonstrated that abnormal miR-21 expression is responsible for glioma invasion by disrupting the negative feedback circuit of Ras/MAPK signaling in glioma cells lacking PTEN under certain tumor microenvironments [28]. We hypothesized that miR-21 would become an invaluable target for inducing radio-sensitivity in glioma cells in response to irradiation, because aberrant miR-21 expression is a common factor under cytotoxic stress, and irradiation-enhanced glioma invasion is observed in PTEN-deficient glioma cells [28], [34]. In this study, we observed a difference in endogenous miR-21 expression in tested malignant glioma cell lines and dose-dependent radiation-induced miR-21 over-expression. The level of endogenous miR-21 expression was proportionately correlated with both miR-21 expression after irradiation and radio-resistance measured by a cytotoxic cell survival assay. The difference in miR-21 expression between PTEN-deficient cells (U373 and U87 cells) and wild-type (WT) PTEN cells (LN18 and LN 428 cells) was clear, and the radio-resistance of PTEN-deficient cells compared to that of WT-PTEN was also evident. Our data suggest that miR-21 expression might not be the sole determining factor for radio-resistance but that radio-resistance by miR-21 was correlated with its expression level after irradiation.

We found that introducing antimiR-21 to PTEN-deficient glioma cells and miR-21 over-expression in WT-PTEN glioma cells led to radio-sensitization and radio-resistance, respectively. Zhou et al. [35] determined that miR-21 knockdown induces apoptosis in both PETN mutant (U251 cells) and WT-PTEN (LN229 cells) cell lines to taxol, despite increased PTEN expression in both cell lines. They explained that decreased expression of epidermal growth factor receptor, cyclin D1, and Bcl-2 by anti-miR-21 was responsible for the blocked G_1_/S transition and observed apoptosis. miR-21 has been demonstrated to target a network of key tumor-suppressor molecules [14]. Thus, it is expected that miR-21 downregulation leads to repression of cell growth, increased apoptosis, and cell cycle arrest in many studies. However, our study is the first report suggesting a direct role of miR-21 in response to radiation of glioma cells involving autophagy. The observed radio-sensitization by anti-miR-21 agrees with previous reports on the anti-apoptotic functions of miR-21. Two reports describing the role of microRNA in the radiation response of malignant glioma cells have been published recently [9], [36]. The mechanism of radio-sensitization was explained in the context of the modulation of apoptosis by down-regulation of Bcl-2 in miR-181a-over-expressing cells [9] and up-regulation of p27 (kip 1) in miR-221/222 knock-down cells [36]. In our study, changes in the cell cycle caused by anti-miR-21 were prominent at the G_2_/M phase, and autophagy-mediated apoptotic cell death occurred after irradiation, although the suppression of PI3K downstream could be a main reason for the radio-sensitization.

### Radiation and DNA Repair in Malignant Gliomas

Although radiation therapy definitely increases the survival of patients with malignant gliomas, it is still far from a cure, and 90% of tumors recur inside the irradiated tumor volume [37], [38]. Among many oncogenic tumor factors contributing to radio-resistance, the role of the PI3K signaling pathway, which is involved in inhibiting apoptosis from chemotherapy or ionizing radiation, has been extensively studied [39]. Additionally, PTEN, a tumor suppressor gene that negatively regulates the PI3K signaling pathway, is inactivated in 40–50% of patients with glioblastomas [40]. Recent studies have revealed a direct relationship between radio-resistance and activated Akt in glioma cells. Basal Akt levels in various malignant glioma cell lines are correlated with radio-resistance [41], and restoration of PTEN in PTEN-deficit glioma cell lines (U251 cells) does not affect radiation-induced DNA foci formation but does affect resolution of the foci with time [32]. The main target of irradiation is DNA [42], and random DSBs have been suggested to be one of the most severe cellular responses [43]. DSBs in DNA after irradiation activate various proteins in complex networks to cope with the induced damage [44]. One of the first cellular responses to DSBs is the formation of γ-H2AX foci [43], [45] at levels proportional to the number of DSBs, and these foci play a critical role in the local recruitment of DNA repair factors [46].

We showed that a potential mechanism for radio-sensitization by anti-miR-21 might be disruption of the DNA damage repair process. Thus, we speculate that miR-21 might be related to repair of DNA damage rather than formation of DNA foci, based on the observation of retained DNA foci at late time points (24 hours after irradiation) in anti-miR-21-treated cells, which showed the same level of γ-H2AX expression at early time points. In our experiments, anti-miR-21-induced persistent DNA foci may have been due to suppression of positive regulators of the PI3K pathway. Numerous reports have indicated that deregulated activation of the RAS/PI3K/AKT pathway in glioblastoma and other carcinomas results in resistance to radiation therapy[17]–[21]. Additionally, synthetic PI3K inhibitors (e.g., wortmannin and LY294002) radio-sensitize cancer cells [17], [22]. We observed a delay in the resolution of DNA foci in LY294002-treated U373 cells after irradiation (Figure S4A), dose-dependent activation of radiation-induced phospho-Akt (ser 473) in the negative-control treated group, and its suppression in the anti-miR-21-treated group. Thus, we suggest that anti-miR-21 slows the repair of DNA damage induced by radiation, presumably by inactivating the PI3K/AKT pathway. Our observation of enhanced autophagy in anti-miR-21-treated cells after irradiation can also be explained partly by inactivation of the PI3K/AKT pathway. As shown earlier, the mammalian target of rapamycin, a potent inhibitor of autophagy, is a down-stream target of Akt, and chemical inhibition of the PI3K/Akt pathway in PTEN-deficit glioma cell lines increases autophagy [47]. However, decrease of autophagy by miR-21 over-expression in LN428 was not associated with concomitant increase of phospho-Akt and anti-miR-21 showed additive effect on autophagy with LY294002 after irradiation in U373 (Supplementary Data). These results suggest that miR-21 modulate autophagy via other than PI3K/AKT pathway.

### Role of Autophagy in Radiation-induced Cell Death

Radiation-induced apoptosis is delayed to days or a week in non-sensitive cell lines or at low dose irradiation levels, whereas in sensitive cell lines or at high doses irradiation induces early apoptosis within a few hours and reaches a plateau before 24 hours [48]. The extent of DNA damage during delayed apoptosis is insufficient to halt cell division, but the remaining unrepaired DNA damage causes cell cycle arrest at the G_2_/M phase, and the accumulated DNA damage causes cell death after aberrant mitosis (post-mitotic apoptosis). Those investigators observed that this transient increase in the G_2_/M population was independent of both caspase-3 activation and changes in mitochondrial membrane potential. Later, Paglin et al. discovered that epithelial cancer cells do not undergo apoptosis after irradiation but accumulate AVOs, indicative of autophagy [26]. These observations of autophagy without apoptosis after radiation were confirmed by Yao et al. in malignant glioma cell lines [27]. They observed that a single-dose of 5–20 Gy irradiation did not induce apoptosis, based on terminal deoxynucleotidyl transferase (TdT)-mediated dUTP nick end labeling staining, whereas autophagy measured by AVO formation was prominent within 1 week [27]. Later, this group observed inhibited autophagy following treatment of radio-sensitized U373 cell lines with 3-MA or bafilomycin-A1 after irradiation [49]. This contrasts with our observation of reversing anti-miR-21-induced radio-sensitization by 3-MA. Although specific conditions determining whether radiation-induced autophagy boost or inhibit radiation-induced cell death are not yet known, it is well understood that on some occasions, autophagy induces death of damaged cells [30], [50]. On other occasions, autophagy plays a protective role rather than programming cell death [51], [52]. Although some molecules responsible for switching autophagy to cell death have been studied, autophagy-induced cell death is largely unknown. Two investigated and accepted facts about autophagy-induced cell death are that it occurs in proportion to the degree of intracellular damage and that autophagy occurs more in apoptosis-defective conditions [53], [54]. In other words, autophagy acts to protect cells under sublethal damage conditions, and it differentially leads to cell death after lethal damage. In a study of inhibited autophagy resulting in radio-sensitization, the authors measured radio-sensitization using a clonogenic assay after irradiation of 2 and 4 Gy [49], and the dose level was sublethal in subconfluent culture condition of Yao et al. and ours to measure the cell cycle, autophagy, and apoptosis. Thus, we postulated that inhibiting autophagy by 3-MA at sublethal irradiation levels could sensitize glioma cells to radiation by eliminating the cytoprotective effect, but inhibition at a lethal dose of 8 Gy showed the reverse of anti-miR-21 induced radio-sensitization in our study, as autophagy contributed to radiation-induced cell death. Thus, whether autophagy acts to protect cells from radiation injury or prompts cells toward radiation-induced cell death is being considered by our laboratory in a future study.

Our observations of increased G_2_/M arrest and enhanced autophagy in anti-miR-21 treated glioma cells are in accordance with so-called “post-mitotic apoptosis”, meaning that cell death occurs late (after at least one cell division). In this context, the reason why the subG_1_ population observed at 48 hours after irradiation failed to show a significant difference, whereas Annexin-V staining, which is supposed to detect earlier apoptosis change, showed a significant increase in apoptosis at the same time lapse in anti-miR-21 treated cells, can be postulated.

Several studies have found that knock down of particular microRNAs induces a change in cell cycle progression by targeting molecules with an essential role in cell cycle regulation [36], [55], [56]. However, those cell cycle changes vary from G_1_ arrest to S phase and increase according to the specific microRNA and combined noxious stimuli. Although we did not test the cell cycle machinery with respect to miR-21 over-expression, anti-miR-21- induced increases in the G_2_/M fraction following radiation could result from increased levels of unrepaired DNA damage based on the observation of retained DNA foci and that the G_2_/M transition and DNA damage level were in accordance with post-mitotic apoptosis.

The main evidence that we do not provide in this study is the specific molecular targets of miR-21 during anti-miR-21-induced radio-sensitivity. It is difficult to determine particular molecular targets because of the complex biological processes in response to radiation. Radiation-dose specificity and cell type dependence in response to radiation have been disclosed through the frantic efforts of many researchers to identify radiation-induced genes or targets from microarray analyses [3], [57], [58]. These gene expression profiles might reflect genetic heterogeneity of solid tumors or different radio-resistance between patients with respect to radiation therapy. However, radiation-induced gene expression is not completely correlated with the corresponding proteins as a whole. Lu et al. suggested that radiation-induced translational control is more a fundamental component of cellular radio-response than transcriptional control based on microarray analysis of both total RNA and polysome-bound RNA in irradiated human brain tumor cells [59]. Taken together, these observations might imply that the radio-resistant trait is maintained by the enormous and vigilant molecular mechanism of the glioma, and that it is necessary to regulate many radiation-induced genes by a handful of regulatory factors such as microRNA. From this point of view, it might be indispensable to identify a link between the radiation-induced signaling network and miR-21 than to provide a miR-21 target [11], [15], [28], [60] for controlling the vast targets of radiation therapy. In other words, we suggest that miR-21 itself is the pivotal target for applying radiation therapy.

We demonstrated that miR-21 down-regulation contributed to the radio-sensitization of glioma cells and that irradiation-induced autophagy was affected by anti-miR-21. Our results describe an important role of miR-21 in radio-resistance of malignant glioma and provide novel therapeutic hints for future treatment of malignant glioma.

## Materials and Methods

### Cell Cultures, γ-irradiation, and Transfection

The U373, U87, LN18 and LN428 cell lines, as well as human glioma cells were obtained from the American Type Culture Collection (Manassas, VA, USA) and grown in Dulbecco′s modified Eagle′s medium (Life Technologies, Inc., Grand Island, NY, USA) supplemented with 10% fetal bovine serum and antibiotics (WelGene, Inc., Dae-gu, Korea). Cell cultures were maintained at 37°C and 5% CO_2_. Cells were irradiated using a Gammacell® 1000 Elite Cesium^137^ source (MDS Nordion, Ottawa, ON, Canada) at a dose rate of 0.063 Gy/sec. The miRIDIAN Hairpin inhibitor (hereafter, anti-miR-21) to has-miR-21 and meridian microRNA Hairpin inhibitor negative control #1 (hereafter, negative control) were purchased from Dharmacon (Chicago, IL, USA) to suppress miR-21, and cells were transfected using the Amaxa Nucleofactor kit (Lonza, Walkersville, MD, USA) in accordance with the classification of the cell lines. Transfection complexes were prepared according to the manufacturer’s instructions and added directly to the cell suspensions to a final concentration of 100 nM with anti-miR-21 and the negative control. Then, the cells in all experimental groups were electroporated. The transfected cells were incubated for a further 48 hours prior to γ-irradiation. We used the miR-21 plasmid and the empty plasmid as a negative control for miR-21 gain-of-function in the LN18 and LN428 cell line as described previously [28]. Transfection methods using two plasmids were identical to the above anti-miR-21 and negative control transfection methods.

### Cytotoxic Cell Survival Assay

A cytotoxicity cell survival curve was obtained after irradiation by determining the relative fraction of survived cells to the unirradiated control, and cell viability was measured by the aulforhodamin-B (SRB) assay [29]. Cells were seeded in 96-well plates at 1500 cells/well and allowed to attach overnight before radiation treatment. Five days after the radiation exposure, the cells were fixed with 50 µl cold 10% trichloroacetic acid and incubated for at least for 1 hour at 4°C. The fixed cells were then washed with distilled water five times and air-dried. The dried cells were stained with 0.4% SRB in 1% acetic acid solution for 30 minutes, washed with 1% acetic acid solution, and air-dried again. Stained SRB, which was uptaked in proportion to cellular protein mass, was resolved with 10 mM unbuffered Tris-base solution (pH 10.5). SRB dye absorption was measured at 540 nm. Cell viability after irradiation was also measured by the colony forming assay. In all experiments, the cells were allowed to stand in 60-mm culture dishes for 24 hours before irradiation. Colonies were stained with the Diff Quick Stain kit (Sysmex, Kobe, Japan) and counted 14 days after irradiation. A cluster of 50 cells or more was scored as a colony, and the surviving fraction was calculated in relation to the untreated control wells.

### MicroRNA-21 Expression Analysis

Total RNA from human cell lines was isolated with Trizol reagent (Invitrogen, Carlsbad, CA, USA) according to the manufacturer’s instructions, except during the isopropyl alcohol precipitation step. Mussel glycogen (20 µg/µl) was added during isopropyl precipitation to reduce the loss of endogenous microRNA. Total RNA was treated with DNase I (Promega, Madison, WI, USA) to remove contamination by genomic DNA and purified using the phenol-chloroform method. For Northern blots, 20 µg of purified total RNA was loaded onto a 15% urea-polyacrylamide gel, and the separated small RNAs were transferred to a super negative charged nylon membrane using a semi-dry transfer machine. Anti-miR-21 (5′-TCAACATCAGTCTGATAAGCTA-3′), as a probe, was synthesized and end-labeled with γ-P^32^-dATP using T4 kinase (Promega, Madison, WI, USA). The membranes were hybridized at 37°C in a hybridization machine for 24 hours. After a final wash step, the membranes were exposed to blue X-ray film at −80°C. 5S rRNA was used as the internal control. For the real-time quantitative polymerase chain reaction (qRT-PCR), we measured miR-21 with a mirVana qRT-PCR detection kit (Ambion, Austin, TX, USA) using the mirVana qRT-PCR primer set for hsa-miR-21 and the internal control (Ambion) in accordance with the manufacturer’s instructions. Relative miR-21 expression was normalized to that of U6 snRNA.

### Analysis of γ-H2AX Foci

Cells were seeded on four-well chamber slides at a density of 1×10^5^ cells per well and incubated overnight at 37°C with 5% CO_2_. All groups of cells were fixed in 3.7% formaldehyde, blocked with 1% BSA/0.2% Triton-X-100 diluted in PBS, and probed with anti-γ-H2AX antibody (Bethyl Laboratories, Inc., Montgomery, TX, USA) followed by a secondary antibody (anti-rabbit Alexa Fluor 488; Invitrogen). After staining with the specific antibody, the chamber slides were counterstained with 4'-6-diamidino-2-phenylindole (DAPI) (Invitrogen) to mark the nuclei. All groups were then assessed for γ-H2AX foci using confocal microscopy (LSM 510 META, Carl-Zeiss, Jena, Germany). Foci were counted in 300 cells per group, and the means and standard error values for foci per cells were calculated for each group using Sigma Stat 3.1 (Systat Software, Inc., San Jose, CA, USA).

### Cell Cycle Analysis

Cells transfected with either antimiR-21 or the negative control were cultured subconfluently on 60-mm dishes and harvested for analysis at 2 days after irradiation. Attached cells were trypsinized, pelleted by centrifugation at 2,500 rpm for 5 min, rinsed with PBS, and repelleted with 1 ml of PBS. The cells were fixed by adding 4 ml of ice-cold 70% ethanol dropwise while vortexing and then incubated at 4°C for at least 1 hour. The fixed cells were then pelleted by centrifugation, rinsed with PBS, and repelleted. The cells were then incubated with 100 µl propidium iodide (50 µg/ml; Sigma-Aldrich, St. Louis, MO, USA) containing 100 µg RNase (1 mg/ml in PBS; Qiagen, Valencia, CA, USA) at 37°C for 1 hour. The cells were finally analyzed by fluorescence-activated cell sorter (BD Biosciences, Franklin Lakes, NJ, USA) with CellQuest software (BD Biosciences) to determine their cell cycle stage.

### Autophagy Measurements using Acridine-orange Staining

Autophagy is the process of protein sequestration after unification with the lysosome, and results in the formation of acidic vesicular organelles (AVOs). These AVOs are red when stained with acridine orange (Sigma-Aldrich). To detect and quantify autophagic activity, cells grown on 60-mm plates were trypsinized, stained, and analyzed by flow cytometry 2 days after irradiation. Briefly, cells were incubated at 37°C for 15 minutes with acridine orange (1 µg/ml) containing medium before trypsinization. Trypsinized, washed, and pelleted cells were resuspended in PBS: complete media (1∶1) until the flow cytometry measurements. The stained cells were analyzed using a fluorescence-activated cell sorter, and data were collected and analyzed using CellQuest software.

### Autophagy Measurements using GFP-LC3

A green fluorescent protein-microtubule-associated protein 1 light chain 3 (GFP-LC3) expression plasmid (a kind gift from Dr. Seiji Kondo [30]) was subcloned into pCDH-EF2-MCS-T2A-Puro, a lentiviral vector for cDNA expression (System Biosciences, Mountain View, CA, USA). U373 cells were transfected with this lentiviral GFP-LC3 vector and expressed GFP-LC3 stably under puromycin selection. Cells were transiently transfected with anti-miR-21 or the negative control, as indicated, and grown on a microscope cover glass. After confirming green fluorescence, the cells, at half confluent density, were irradiated at 8 Gy. Two days later, the cells on glass were fixed with ethanol and mounted with DAPI to fluorescently counterstain the nuclei. Cells were scored as undergoing autophagy if GFP-LC3 granules or punctuated spots were observed under a fluorescence microscope. The percentage of cells undergoing autophagy was averaged from 10 low power fields (×200) and calculated from the ratio of autophagic cells to normal cells bearing GFP-LC3 fluorescence.

### Western Blotting

Cells were lysed in lysis buffer (20 mM Tris-HCl pH 7.4, 150 mM NaCl, 1% Triton X-100, 0.1 mM EDTA, 1 mM EGTA, 2 mM sodium orthovanadate, 2 mM NaF, and Complete™ Protease Inhibitor Mix [Roche Applied Science, Mannheim, Germany]) for 20 min on ice and cleared by centrifugation at 12,000 rpm and 4°C. Proteins were resolved on a 10% SDS-PAGE gel, transferred onto nitrocellulose membranes, blocked with 5% nonfat dry milk in TBST (10 mM Tris-HCl pH 7.5, 100 mM NaCl, and 0.05% Tween 20) followed by incubation with a primary antibody [anti-LC3B antibody (Novus Biologicals, Littleton, CO, USA), total and anti-phospho-AKT (Ser473) antibody (Cell Signaling Biotechnology, Beverly, MA, USA), anti-Rad51 antibody (abcam), and anti-β-actin antibody (Sigma-Aldrich)]. Blots were washed and incubated with horseradish peroxidase-conjugated secondary antibody. Antibody complexes were visualized using an enhanced chemiluminescence-Western blotting detection system (Thermo Fisher Scientific, Inc., Rockford, IL, USA).

### Analysis of Apoptosis with Annexin-V FITC

Cells were treated using the same conditions as used for the autophagy measurements. Plated cells were trypsinized, washed twice with PBS, and then resuspended at a concentration of 1×10^6^ cells/ml in binding buffer (10 mM HEPES/NaOH, pH 7.4, 140 mM NaCl, 2.5 mM CaCl_2_). A 10 µl aliquot of Annexin V-FITC (BD Biosciences, Franklin Lakes, NJ, USA) and 5 µl of propidium iodide (Sigma-Aldrich), at a concentration of 50 µg/ml, were added to 100 µl of cell suspension following the manufacturer’s instructions, and the cells were incubated at room temperature for 15 minutes in the dark. The stained cells were analyzed using a fluorescence-activated cell sorter, and data were collected and analyzed using CellQuest software.

### Statistics

Data were initially evaluated based on a normal distribution. Statistical significances among groups were tested using Sigma Stat 3.1 (Systat Software, Inc., San Jose, CA, USA) and the unpaired Student’s *t*-test. Differences were considered significant with *p-*values <0.05.

## Supporting Information

Figure S1 **The relative expression of miR-21 before (empty column) and after (black column) γ-irradiation in various glioma cell lines according to the PTEN status** (wild type (LN18, LN229, and LN428) vs. mutant type (A172, U251, U373, and U87) is illustrated by real-time PCR data. Each error bar indicates the standard deviation of three independent experiments.
(TIF)

Figure S2 **The relative knock-down of miR-21 after shRNA transfection.** For U373 (A) and U87 cells (B), real-time PCR was used to assay the miR-21 expression level. For LN18 cells (C), northern blot was used to identify the miR-21 over-expression.
(TIF)

Figure S3 **The radio-sensitivity of glioma cells according to the modulation of miR-21 measured by colony forming assay.** After exposure to the indicated level of ionizing irradiation, radio-resistance was analyzed using a clonogenic survival assay at 10–14 days and the effect of miR-21 modulation is illustrated for each cell line. For U373 (A) and U87 cells (B), anti-miR-21 was transfected while for LN 18 (C), miR-21 was over-expressed (See Materials & Methods for detail). Each error bar indicates the standard deviation of three independent experiments.
(TIF)

Figure S4 **Supportive data of anti-miR-21 inhibition of DNA DSB repair through phospho-Akt-suppression in U373 cells.** (A) The effect of suppression of DNA foci resolution by PI3K inhibitor (LY294002) in U373 glioma cells was illustrated by immunofluorescence (blue-DAPI, green-γ-H2AX of DNA foci). The γ-H2AX of DNA foci after irradiation was sustained at 24 hours following irradiation with PI3K inhibitor (LY294002). (B) Down-regulation of Rad51, which is known to be an up-regulated a DNA DSB repair protein after radiation injury, by anti-miR-21 transfection is demonstrated with Western blot. (C) Quantitative analysis of relative phospho-Akt expression after irradiation standardized to negative control transfected, un-irradiated level using image analysis software (See details in Result section).
(TIF)

Figure S5 **Influence of miR-21 over-expression on phospho-Akt in PTEN wild type glioma cells (LN428).** (A) Marginal increase of phospho-Akt after irradiation in negative control group is not observed in miR-21 over-expression group by Western blot. (B) Quantitative analysis using image software shows no phospho-Akt elevation in miR-21 over-expression group after irradiation. Each error bar indicates the standard error mean of three independent experiments.
(TIF)

Figure S6 **Influence of inhibition of Akt phosphorylation by LY294002 in anti-miR-21 induced radiosensitivity or miR-21 over-expression induced radioresistance.** (A) In U373 cells (PTEN non-functional), LY294002 showed anti-apoptotic effect in the control group but revealed ‘a little effect on radiosensitivity’ in miR-21 knock down cells. (B) In LN428 cells (PTEN wild type), LY294002 did not show any discernible difference of radiosensitivity either in the control groups or in the miR-21 over-expressed groups. Each error bar indicates the standard deviation of three independent experiments.
(TIF)

Figure S7 **Supportive data illustrating additive role of miR-21 and phospho-Akt in radiation-induced autophagy.** (A) The inhibition of radiation-induced autophagy by 3-MA is measured by by flow cytometry measurement of acidic vesicular organelles (AVO). This inhibition neutralized anti-miR21-induced augmented apoptosis after irradiation (See figure 6F in the manuscript). (B) miR-21 over-expression inhibited autophagy after irradiation in PTEN wild type cells (LN428), which showed no increase of phospho-Akt. This inhibition is vice versa of anti-miR-21 effect on autophagy in PTEN mutant type cells. (C) LY29004 and anti-miR-21 increased autophagy after irradiation in a synergistic manner. We suggest that miR-21 can modulate autophagy not only thorough PI3K/AKT pathway but also via other pathway. Each error bar indicates the standard error mean of three independent experiments.
(TIF)
