# Supplementary figures and images for: Silencing of MicroRNA-21 Confers Radio-Sensitivity through Inhibition of the PI3K/AKT Pathway and Enhancing Autophagy in Malignant Glioma Cell Lines
Source: PLoS One. 2012 Oct 15;7(10):e47449. doi: 10.1371/journal.pone.0047449 (PMC3471817; doi:10.1371/journal.pone.0047449)

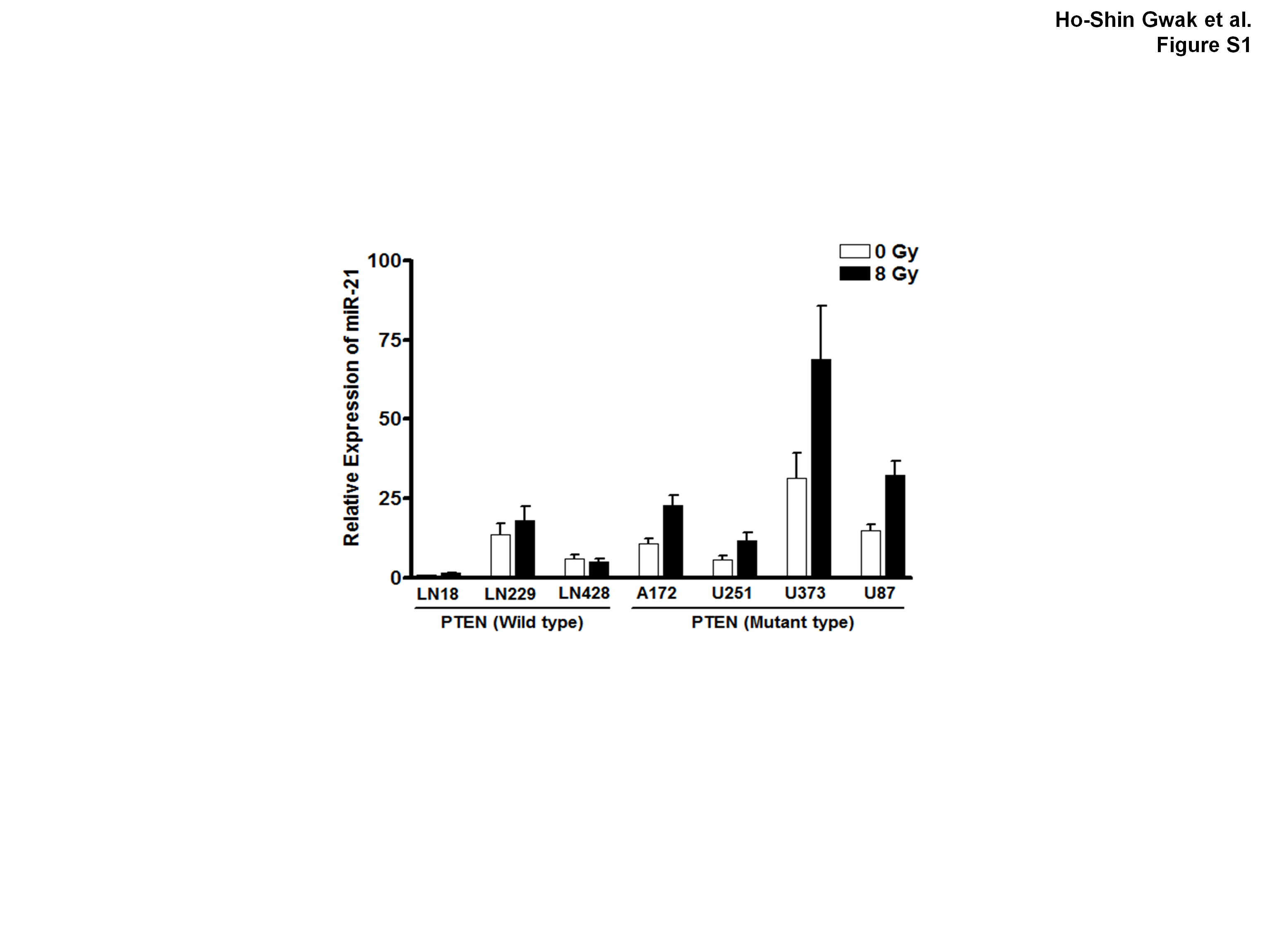

Supplement: Figure S1 — The relative expression of miR-21 before (empty column) and after (black column) γ-irradiation in various glioma cell lines according to the PTEN status (wild type (LN18, LN229, and LN428) vs. mutant type (A172, U251, U373, and U87) is illustrated by real-time PCR data. Each error bar indicates the standard deviation of three independent experiments. (TIF) [file pone.0047449.s001.tif]

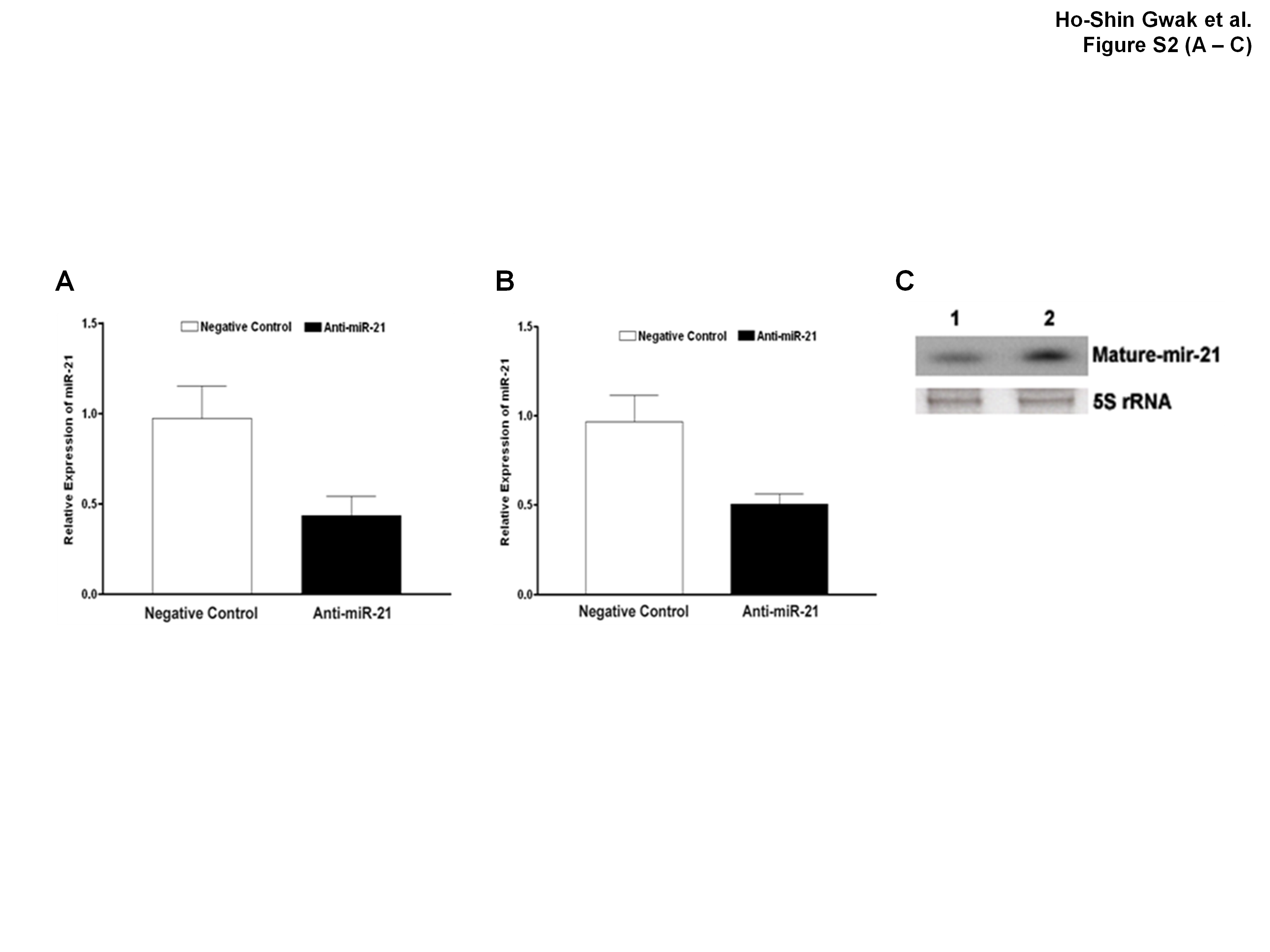

Supplement: Figure S2 — The relative knock-down of miR-21 after shRNA transfection. For U373 (A) and U87 cells (B), real-time PCR was used to assay the miR-21 expression level. For LN18 cells (C), northern blot was used to identify the miR-21 over-expression. (TIF) [file pone.0047449.s002.tif]

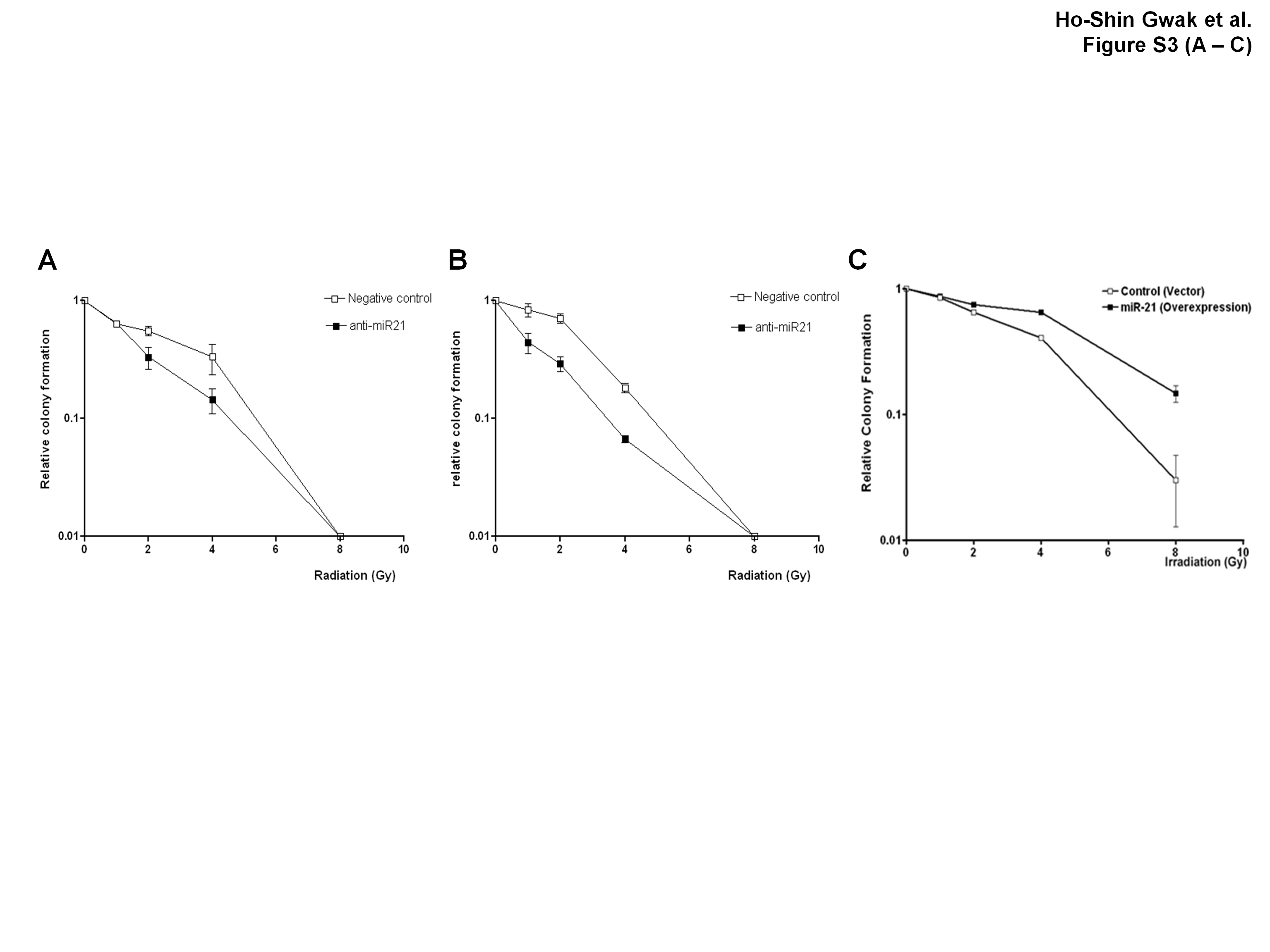

Supplement: Figure S3 — The radio-sensitivity of glioma cells according to the modulation of miR-21 measured by colony forming assay. After exposure to the indicated level of ionizing irradiation, radio-resistance was analyzed using a clonogenic survival assay at 10–14 days and the effect of miR-21 modulation is illustrated for each cell line. For U373 (A) and U87 cells (B), anti-miR-21 was transfected while for LN 18 (C), miR-21 was over-expressed (See Materials & Methods for detail). Each error bar indicates the standard deviation of three independent experiments. (TIF) [file pone.0047449.s003.tif]

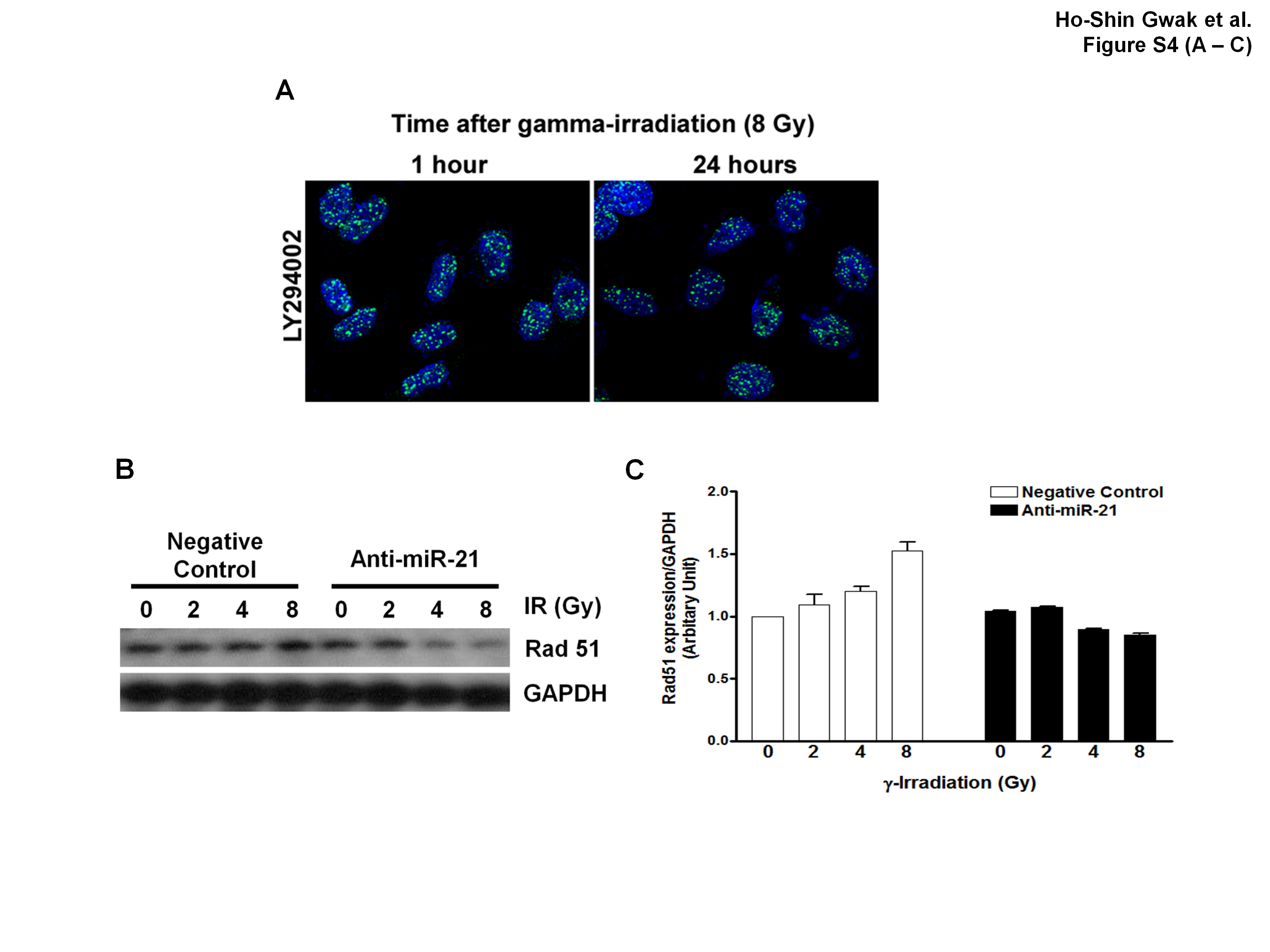

Supplement: Figure S4 — Supportive data of anti-miR-21 inhibition of DNA DSB repair through phospho-Akt-suppression in U373 cells. (A) The effect of suppression of DNA foci resolution by PI3K inhibitor (LY294002) in U373 glioma cells was illustrated by immunofluorescence (blue-DAPI, green-γ-H2AX of DNA foci). The γ-H2AX of DNA foci after irradiation was sustained at 24 hours following irradiation with PI3K inhibitor (LY294002). (B) Down-regulation of Rad51, which is known to be an up-regulated a DNA DSB repair protein after radiation injury, by anti-miR-21 transfection is demonstrated with Western blot. (C) Quantitative analysis of relative phospho-Akt expression after irradiation standardized to negative control transfected, un-irradiated level using image analysis software (See details in Result section). (TIF) [file pone.0047449.s004.tif]

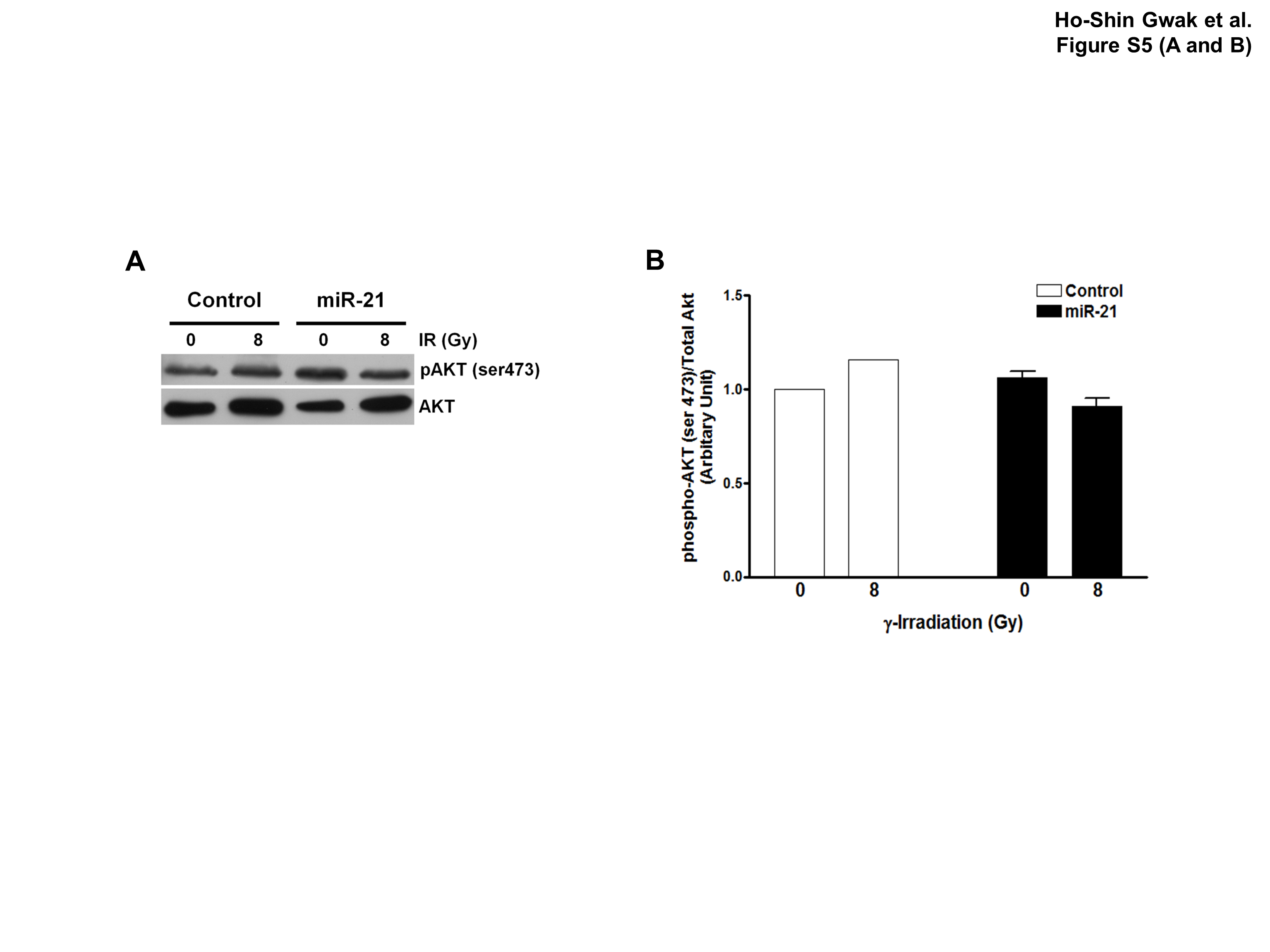

Supplement: Figure S5 — Influence of miR-21 over-expression on phospho-Akt in PTEN wild type glioma cells (LN428). (A) Marginal increase of phospho-Akt after irradiation in negative control group is not observed in miR-21 over-expression group by Western blot. (B) Quantitative analysis using image software shows no phospho-Akt elevation in miR-21 over-expression group after irradiation. Each error bar indicates the standard error mean of three independent experiments. (TIF) [file pone.0047449.s005.tif]

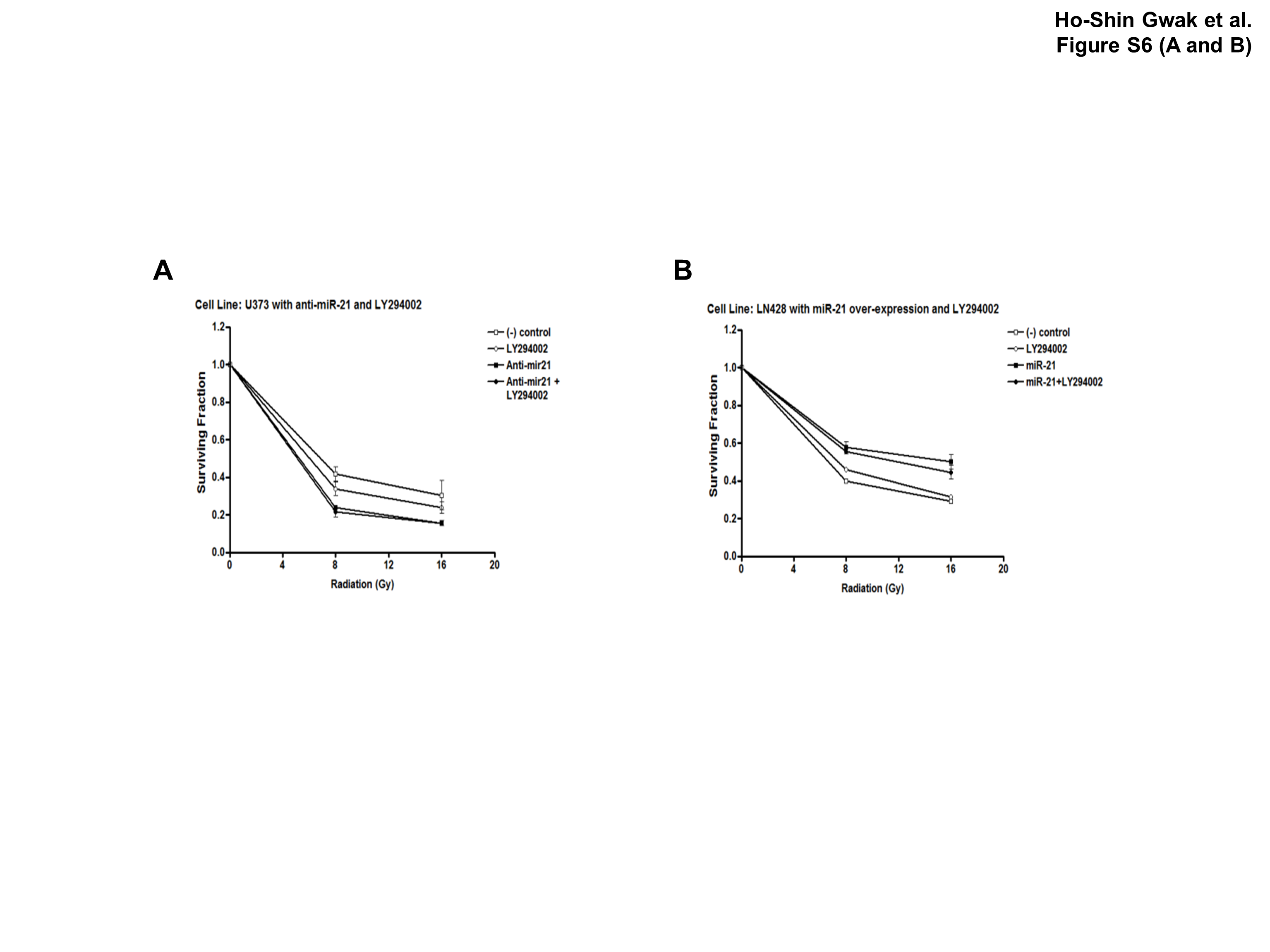

Supplement: Figure S6 — Influence of inhibition of Akt phosphorylation by LY294002 in anti-miR-21 induced radiosensitivity or miR-21 over-expression induced radioresistance. (A) In U373 cells (PTEN non-functional), LY294002 showed anti-apoptotic effect in the control group but revealed ‘a little effect on radiosensitivity’ in miR-21 knock down cells. (B) In LN428 cells (PTEN wild type), LY294002 did not show any discernible difference of radiosensitivity either in the control groups or in the miR-21 over-expressed groups. Each error bar indicates the standard deviation of three independent experiments. (TIF) [file pone.0047449.s006.tif]

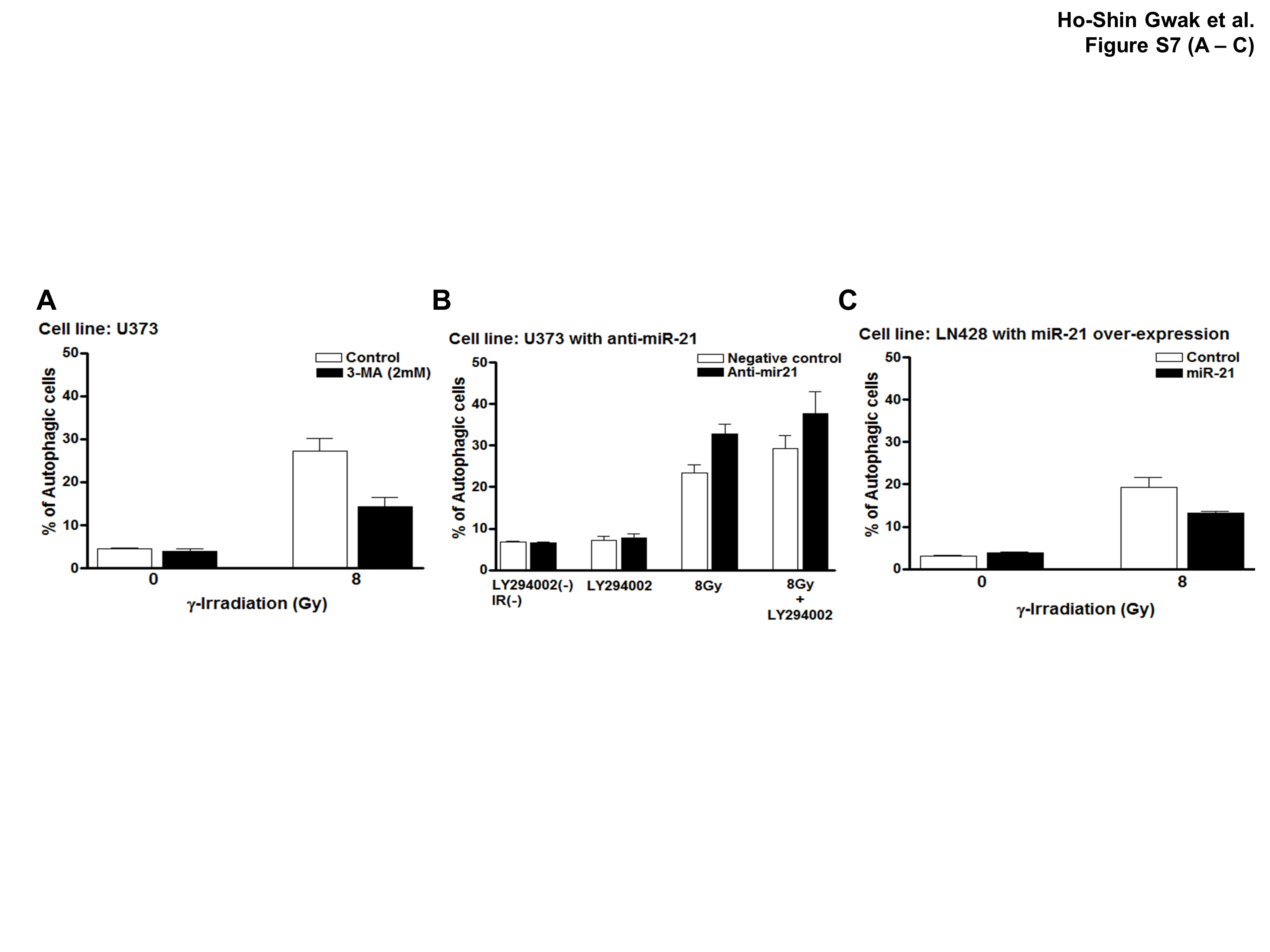

Supplement: Figure S7 — Supportive data illustrating additive role of miR-21 and phospho-Akt in radiation-induced autophagy. (A) The inhibition of radiation-induced autophagy by 3-MA is measured by by flow cytometry measurement of acidic vesicular organelles (AVO). This inhibition neutralized anti-miR21-induced augmented apoptosis after irradiation (See figure 6F in the manuscript). (B) miR-21 over-expression inhibited autophagy after irradiation in PTEN wild type cells (LN428), which showed no increase of phospho-Akt. This inhibition is vice versa of anti-miR-21 effect on autophagy in PTEN mutant type cells. (C) LY29004 and anti-miR-21 increased autophagy after irradiation in a synergistic manner. We suggest that miR-21 can modulate autophagy not only thorough PI3K/AKT pathway but also via other pathway. Each error bar indicates the standard error mean of three independent experiments. (TIF) [file pone.0047449.s007.tif]
